# Synergistic Engineering of CoO/MnO Heterostructures Integrated with Nitrogen-Doped Carbon Nanofibers for Lithium-Ion Batteries

**DOI:** 10.3390/molecules29102228

**Published:** 2024-05-09

**Authors:** Donglei Guo, Yaya Xu, Jiaqi Xu, Kailong Guo, Naiteng Wu, Ang Cao, Guilong Liu, Xianming Liu

**Affiliations:** 1 Key Laboratory of Function-Oriented Porous Materials, College of Chemistry and Chemical Engineering, Luoyang Normal University, Luoyang 471934, China; gdl0594@163.com (D.G.); xuyayachonga@163.com (Y.X.); 15397973347@163.com (K.G.); wunaiteng@gmail.com (N.W.); 2 Department of Physics, Technical University of Denmark, 2800 Lyngby, Denmark; angc@dtu.dk

**Keywords:** lithium-ion battery, nitrogen-doped carbon nanofibers, heterostructures, density functional theory

## Abstract

The integration of heterostructures within electrode materials is pivotal for enhancing electron and Li-ion diffusion kinetics. In this study, we synthesized CoO/MnO heterostructures to enhance the electrochemical performance of MnO using a straightforward electrostatic spinning technique followed by a meticulously controlled carbonization process, which results in embedding heterostructured CoO/MnO nanoparticles within porous nitrogen-doped carbon nanofibers (CoO/MnO/NC). As confirmed by density functional theory calculations and experimental results, CoO/MnO heterostructures play a significant role in promoting Li^+^ ion and charge transfer, improving electronic conductivity, and reducing the adsorption energy. The accelerated electron and Li-ion diffusion kinetics, coupled with the porous nitrogen-doped carbon nanofiber structure, contribute to the exceptional electrochemical performance of the CoO/MnO/NC electrode. Specifically, the as-prepared CoO/MnO/NC exhibits a high reversible specific capacity of 936 mA h g^−1^ at 0.1 A g^−1^ after 200 cycles and an excellent high-rate capacity of 560 mA h g^−1^ at 5 A g^−1^, positioning it as a competitive anode material for lithium-ion batteries. This study underscores the critical role of electronic and Li-ion regulation facilitated by heterostructures, offering a promising pathway for designing transition metal oxide-based anode materials with high performances for lithium-ion batteries.

## 1. Introduction

Lithium-ion batteries (LIBs) have become indispensable power sources in various electronic devices and electric vehicles due to their eco-friendliness, remarkable energy and power density, and long-term durability [1,2,3]. Nevertheless, the current mainstream anode material, graphite, falls short of meeting the requirements of modern economies due to its limited theoretical specific capacity (372 mA h g^−1^) and low-rate performance [4,5,6]. Consequently, there is a pressing need to explore alternative materials, and transition metal oxides (TMOs) have emerged as promising candidates due to their high theoretical specific capacity, operating via a conversion mechanism [7,8,9]. Among these TMOs, manganese oxide (MnO) holds particular promise as an anode material, boasting a theoretical specific capacity of 756 mA h g^−1^, along with its affordability, non-toxicity, low hysteretic potential, and environmental compatibility [10,11]. However, like many other TMOs, MnO faces several challenges, including poor conductivity and significant volume expansion during the Li^+^ insertion/extraction process, which can lead to sluggish reaction kinetics and electrode pulverization during repeated lithium storage cycles [12,13]. Moreover, the safety and stability of the anode materials of LIBs continue to be set by their inherent structure and characteristics, especially in terms of cyclability and stress tests when used in a variety of electronics and electric vehicles [14,15]. Thus, addressing these challenges is crucial for the practical implementation of MnO-based anodes in LIBs.

Recently, a multitude of effective strategies has been developed to address the above issues, including surface coatings [16,17], the construction of porous nanostructured materials [18,19], and the introduction of heteroatoms [20,21]. For instance, Zhang et al. reported the coupled MnO coated with Co-decorated N-doped carbon (NC/Co-MnO) [16], which exhibited exceptional cycling performance and high-rate capacity. The NC/Co layer can effectively mitigate the volume change in the MnO electrode and facilitate the electron transport pathways. Song et al. synthesized the 3D interconnected porous MnO/C composite materials through the carbonization of aerogels [19], which offered outstanding electronic conductivity and efficient electron/ion transport channels, showing a high specific capacity and remarkable rate capability. Pan et al. synthesized the Ni/MnO porous microspheres, which exhibited superior electrochemical properties [22].

Despite these advancements, the sluggish reaction kinetics persist as a limitation for MnO-based lithium storage. Recently, constructing heterostructures by coupling compounds with differing properties has emerged as an effective strategy for enhancing the Li-ion diffusion kinetics of electrode materials through electronic structure modulation [23,24,25,26,27]. For example, Wu et al. constructed MnO@MnS heterostructures confined within pyrolytic carbon microspheres, demonstrating high-rate capacities (608 mA h g^−1^ at 3.2 A g^−1^) and stable cycling performances (522 mA h g^−1^ under 3.0 A g^−1^ over 2000 cycles) [23]. Bao et al. synthesized MnO/MnSe@NC heterostructure nanoparticles through in situ calcination and selenization [26], which not only facilitated Li^+^/Na^+^ diffusion but also shortened the ion diffusion path, resulting in excellent Li^+^/Na^+^ storage performance. These heterostructures exhibit tunable electronic properties, leading to improved dynamics and the structural stability of electrode materials. Additionally, the heterostructure nanomaterials with interfacial electric fields can significantly improve the electrochemistry kinetics and enhance lithium storage properties [28,29].

Herein, we proposed a facile strategy to improve the electrochemical performance of electrode materials by hybridizing the CoO/MnO heterostructures with porous nitrogen-doped carbon nanofibers (CoO/MnO/NC). The porous nitrogen-doped carbon nanofibers can improve electrical conductivity, provide channels for fast charge transport, and promote the structural stability of CoO/MnO/NC. The incorporation of heterostructures with interfacial electric fields plays a pivotal role in significantly boosting electronic conductivity, reducing Li adsorption energy, and expediting the electrochemical kinetics of CoO/MnO/NC. Capitalizing on these advantages, the CoO/MnO/NC electrode demonstrates exceptional electrochemical performance, exhibiting a highly reversible specific capacity of 936 mA h g^−1^ at 0.1 A g^−1^ after 200 cycles and an impressive high-rate capacity of 560 mA h g^−1^ at 5 A g^−1^ as an anode material for LIBs. This research provides valuable insights into the regulation of electron structure by heterostructures to improve the electrochemical performance of TMO-based electrode materials for LIBs.

## 2. Results and Discussion

### 2.1. Synthesis and Characterization of CoO/MnO/NC

**Figure 1:**
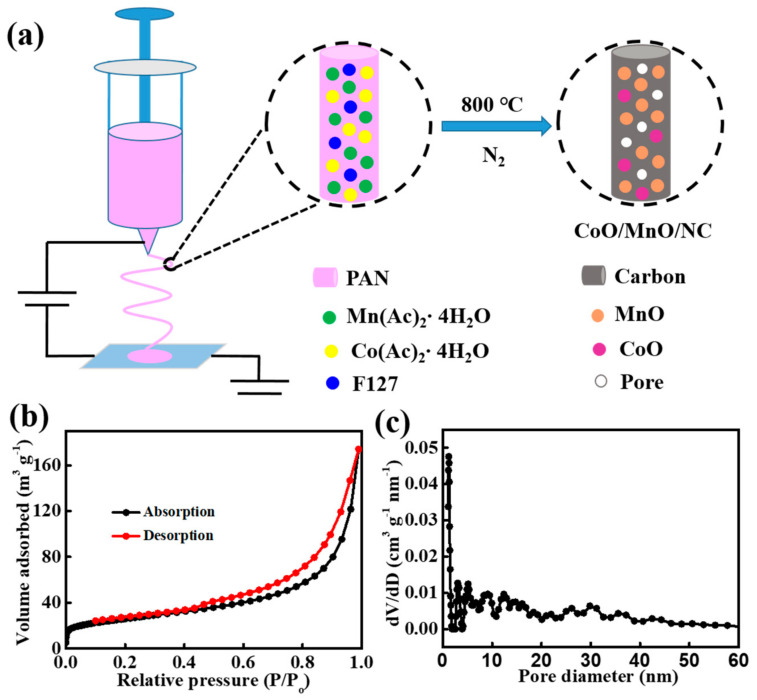
(**a**) Schematic illustration of the synthesis procedure of CoO/MnO/NC heterostructures. (**b**) N_2_ adsorption/desorption isotherm and (**c**) pore size distribution of CoO/MnO/NC according to the NLDFT model.

The CoO/MnO heterostructures with the porous nitrogen-doped carbon (CoO/MnO/NC) nanofibers were synthesized through the electrospinning technique and high-temperature carbonization, and the schematic synthetic process was illustrated in Figure 1a. In brief, the quality of homogeneous dispersions by mixing all precursors is crucial to obtaining polymer nanofibers with a uniform diameter. The as-electrospun polymer nanofibers were initially stabilized and pre-oxidized at 230 °C for 8 h under air atmosphere. The heterostructured CoO/MnO/NC nanofibers were finally obtained via subsequent carbonization at 800 °C for 2 h under a nitrogen atmosphere, and the porous structure was caused by the generated gas from decomposing the polymers. The high porosity of CoO/MnO/NC nanofibers was evaluated via the nitrogen adsorption/desorption isotherm in accordance with the Brunauer–Emmette–Teller (BET) theory, as shown in Figure 1b. The specific surface area of CoO/MnO/NC nanofibers is about 177.5 m^2^ g^−1^, and the average pore size distribution is about 3 nm (Figure 1c). This porous structure can facilitate Li-ion transportation, prevent the aggregation of metal oxides, and restrict volume expansion during charge/discharge processes [30,31].

**Figure 2:**
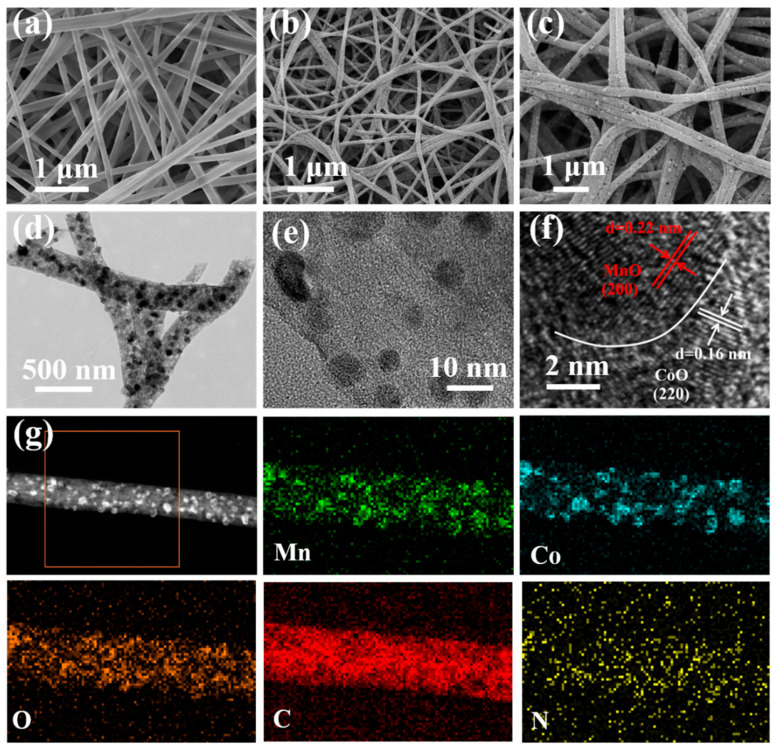
(**a**) SEM image of the pristine polymer nanofibers. (**b**,**c**) SEM images, (**d**,**e**) TEM images, and (**f**) HR-TEM image of CoO/MnO/NC. (**g**) The HAADF-STEM image and element mapping of CoO/MnO/NC.

The morphological characteristics of CoO/MnO/NC nanofibers were evaluated. Figure 2a shows the SEM image of pristine polymer nanofibers with a smooth surface and cross-linked morphology. After stabilization and carbonization, the surface roughness of CoO/NC, MnO/NC, and CoO/MnO/NC nanofibers is increased in contrast to pristine polymer nanofibers (Figure S1). CoO/MnO/NC nanofibers exhibit a rough and continuous surface (Figure 2b,c), and the pores on the surface of carbon nanofibers are a result of the generated gas during the polymer decomposition process. The transition electron microscopy (TEM) technique was further applied to reveal the morphological structure of CoO/MnO/NC nanofibers. It can be found that CoO/MnO nanoparticles are uniformly embedded in the porous carbon framework (Figure 2d). The average size of the metal oxide nanoparticles is about 4–8 nm, and no aggregation phenomena can be observed in Figure 2e. The high-resolution TEM (HR-TEM) image is shown in Figure 2f, and two lattice fringes with a d-spacing of 0.22 and 0.16 nm correspond to the characteristic (200) plane of MnO and (220) plane of CoO. Moreover, the selected area electron diffraction (SAED) pattern shows that the diffraction rings correspond to the MnO and CoO phases, revealing the polycrystalline feature of CoO/MnO/NC heterostructures (Figure S2), while the phase boundary between MnO and CoO implies the formation of heterogeneous interfaces. High-angle annular dark field-scanning transmission electron microscopy (HAADF-STEM) and element mapping images demonstrate the uniform distribution of Mn, Co, O, C, and N elements in CoO/MnO/NC nanofibers (Figure 2g).

**Figure 3:**
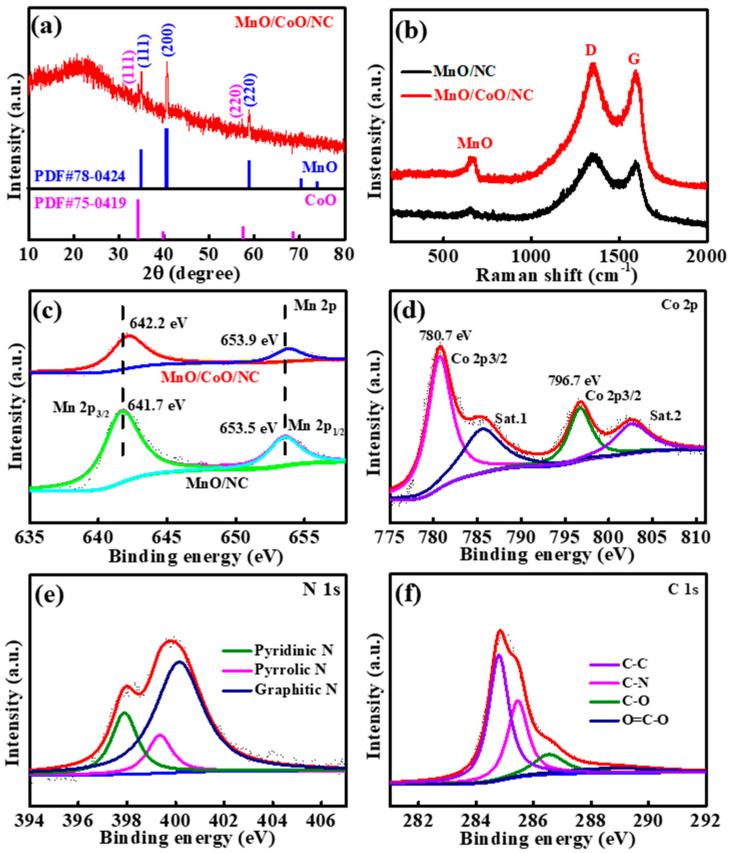
(**a**) XRD pattern of CoO/MnO/NC. (**b**) Raman spectrum of CoO/MnO/NC and MnO/NC. (**c**) High-resolution XPS spectra for the Mn 2p of CoO/MnO/NC and MnO/NC. (**d**–**f**) High-resolution XPS spectra for the Co 2p, N 1s, and C 1s of CoO/MnO/NC.

To evaluate the crystal structure of CoO/MnO/NC nanofibers, the X-ray diffraction (XRD) technique was employed in Figure 3a. The apparent characteristic diffraction peaks correspond to the typical reflection of CoO (PDF#75-0419) and MnO (PDF#78-0424), confirming the successful construction of CoO/MnO/NC heterostructures. The defective sites of CoO/MnO/NC nanofibers were investigated using the Raman spectra shown in Figure 3b, and the dominant peaks at approximately 1600 and 1360 cm^−1^ correspond to the G and D bands of the carbon. The intensity ratio (IG/ID) of CoO/MnO/NC is 0.97, which is higher than that of MnO/NC (0.935), indicating a relatively higher degree of graphitization for CoO/MnO/NC nanofibers [32]. The additional peak at around 536 cm^−1^ corresponds to MnO [26,33]. X-ray photoelectron spectroscopy (XPS) analysis was applied to reveal the surface chemical composition of CoO/MnO/NC, and the characteristic peaks correspond to the Mn, Co, O, C, and N elements in Figure S3. Figure 3c shows the high-resolution XPS spectra of Mn 3d for CoO/MnO/NC and MnO/NC, and two fitted peaks at 641.7 and 653.5 eV correspond to the Mn 2p_3/2_ and Mn 2p_1/2_ of MnO [26,34]. However, for CoO/MnO/NC, the fitting peaks are observed at 642.2 and 653.9 eV due to the heterostructures of CoO/MnO/NC. The high-resolution Co 2p spectrum of CoO/MnO/NC is shown in Figure 3d, and two primary peaks at 780.7 and 796.7 eV are attributed to the Co 2p_3/2_ and Co 2p_1/2_ of the spin-orbital of Co 2p, respectively [35], while the two weak peaks at 786.0 and 802.5 eV correspond to the satellite peaks of Co 2p. For the high-resolution N 1s spectrum of CoO/MnO/NC in Figure 3e, the fitted peaks at 400.1, 399.4, and 397.9 eV correspond to graphitic-N, pyridinic-N, and pyrrolic-N, respectively [36,37]. The C 1s peaks can be deconvoluted into four peaks at 285.5, 284.7, 286.5, and 288.7 eV and correspond to the C-N, C-C, C-O, and O=C-O bonds in Figure 3f, respectively [38,39], and the existence of the C-N bond implies that the heteroatom N is successfully doped into carbon nanofibers. For the O 1s spectra in Figure S3b, the deconvoluted peaks at 529.2, 530.9, and 532.1 eV correspond to the O=C-O, O-C-O, and metal-O bonds [23,26].

### 2.2. Electrocatalytic Performance

**Figure 4:**
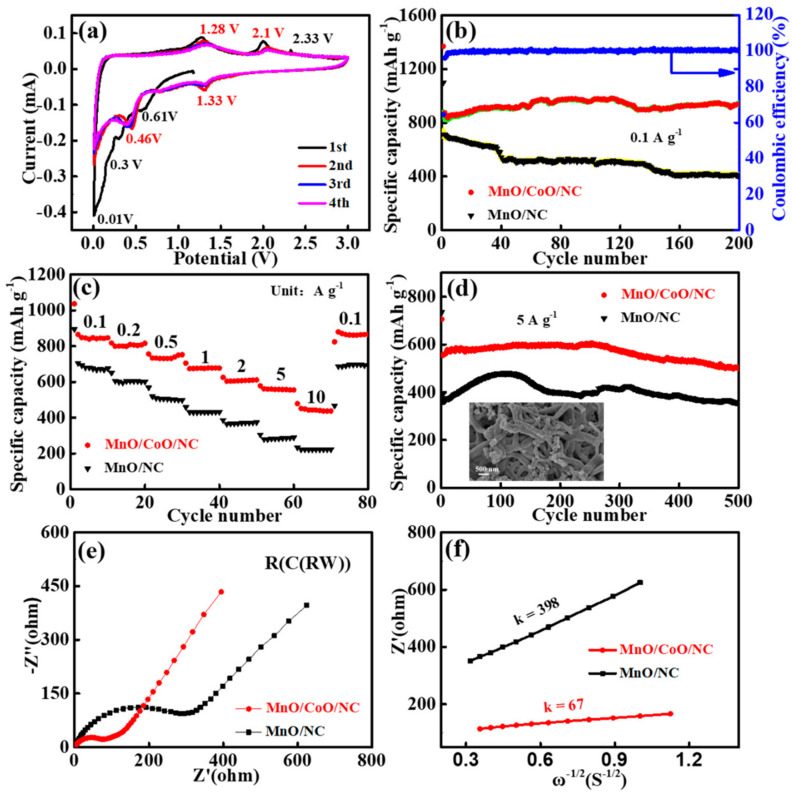
(**a**) CV curves of CoO/MnO/NC. (**b**–**d**) Cyclic stability, rate performance, and cycling performance of CoO/MnO/NC and MnO/NC. The inset in d is the SEM image of the CoO/MnO/NC electrode after cycling for 500 cycles. (**e**) EIS curves of CoO/MnO/NC and MnO/NC. (**f**) The relationship between Z’ and ω^−1/2^ of CoO/MnO/NC and MnO/NC.

Due to the merit of the heterostructure and porous morphology, the electrochemical lithium storage behavior of CoO/MnO/NC electrode materials was investigated using CR2032-type coin cells. Figure 4a depicts the cyclic voltammetry (CV) curves of the CoO/MnO/NC heterostructures at a scan rate of 0.1 mV s^−1^. For the first cycle, an irreversible peak located at 0.61 V can be attributed to the decomposition of electrolytes and the formation of solid electrolyte interphase (SEI) films [26]. Two couples of redox peaks at 0.46/1.28 V and 1.33/2.1 V can be observed, attributed to the conversion reaction process of MnO/CoO to metallic Mn/Co (MnO + 2Li^+^ + 2e^−^ ↔ Mn + Li_2_O, CoO + 2Li^+^ + 2e^−^ ↔ Co + Li_2_O) like many other TMOs [23,39,40,41]. It is worth noting that the observed peak at 2.33 V in the first cycle disappeared in subsequent cycles, which may be caused by a decrease in the overpotential of Mn^2+^ converted to Mn^3+^ or Mn^4+^ and the formation of higher-oxidation-state manganese [26,42]. In addition, the CV curves of the subsequent four cycles are well overlapped, implying the highly reversible behavior of CoO/MnO/NC.

A comparative analysis of CoO/MnO/NC with various Mn:Co ratios reveals that CoO/MnO/NC with a molar ratio of 4:1 (Mn:Co) shows the optimal cycling performance and specific discharge capacity (Figure S4). Figure 4b illustrates the cyclic stability comparison between CoO/MnO/NC and MnO/NC. CoO/MnO/NC exhibited a discharge capacity of 936 mA h g^−1^ after 200 cycles, which is higher than that of MnO/NC (406 mA h g^−1^). Notably, the specific capacity of the CoO/MnO/NC electrode after 200 cycles was higher than the theoretical capacity of MnO (756 mA h g^−1^). This phenomenon can be explained by the electrode activation process, especially for electrode materials with the porous nitrogen-doped carbon framework and large surface areas, which offered extra active sites for Li^+^ storage during discharge/charge processes [12,42,43]. The galvanostatic discharge/charge voltage profiles of CoO/MnO/NC at 0.1 A g^−1^ are presented in Figure S5, exhibiting consistent voltage plateaus with the CV curves. The initial discharge/charge reversible specific capacities of CoO/MnO/NC are 1356 and 865 mA h g^−1^, respectively, and the initial Coulombic efficiency (ICE) is 63.8%. The relatively low ICE can be attributed to the formation of the SEI film, the large specific surface area, and the irreversible insertion of lithium ions [44,45]. Furthermore, a comparison of cyclic stability among CoO/MnO/NC, MnO/NC, and CoO/NC demonstrates that CoO/MnO/NC exhibits the best cyclic performance (Figure S6). The evaluation of rate performances reveals a superior discharge capacity of CoO/MnO/NC compared to MnO/NC at different current densities (Figure 4c). The average specific discharge capacities are 845, 801, 732, 674, 582, 560, and 442 mA h g^−1^ from 0.1 to 10 A g^−1^, which are higher than those of MnO/NC, demonstrating its excellent reversibility. Notably, CoO/MnO/NC was able to recover a discharge capacity of 880 mA h g^−1^ upon returning to a current density of 0.1 A g^−1^. In order to investigate the cyclic stability of CoO/MnO/NC and MnO/NC, the cycling performance was determined at 5 A g^−1^ for 500 cycles in Figure 4d, and CoO/MnO/NC shows the remaining specific discharge capacity of 497 mA h g^−1^ after 500 cycles. The inset image is the SEM of the CoO/MnO/NC electrode after cycling for 500 cycles. As can be clearly seen, the interconnected nanofiber structures were kept well, indicating that the CoO/MnO/NC electrode has a stable structure. The excellent lithium storage performance of CoO/MnO/NC can be attributed to its porous morphology and the formation of heterostructures at the interface.

Electrochemical impedance spectroscopy (EIS) was carried out to further analyze the electrochemistry kinetics process shown in Figure 4e. The Nyquist plots show the electrochemistry relative to the equivalent circuit of R(C(RW)), and the charge transfer resistance (*R*_ct_) of CoO/MnO/NC (75 Ω) is lower in comparison with MnO/NC (296 Ω), indicating that CoO/MnO/NC exhibits faster charge transfers and ion diffusivity [46]. Furthermore, the Li-ion diffusion coefficient (*D*_Li+_) of the samples can be calculated using Equations (1) and (2) [47,48]:

*D*_Li+_ = *R*^2^
*T*^2^/(*2A*^2^
*F*^4^
*n*^4^
*C*^2^
*σ*^2^) (1)

*Z*’ = *R*_s_ + *R*_ct_ + *R*_f_ +*σω*^−1/2^ (2)

where *R* is the gas constant, *A* is the surface area of the electrode, *T* is the absolute temperature, *n* is the number of electrons per molecule during the reaction, *F* is the Faraday constant, and *C* is the concentration of Li-ion. The simulation of the Warburg factor (*σ*) can be calculated using the slope in the fitting line of the *Z*’ vs. *ω*^−1/2^ plots (Figure 4f). The slope value of CoO/MnO/NC (k = 67) is about six times smaller than that of MnO/NC (k = 398). Moreover, the *D*_Li+_ value of CoO/MnO/NC was calculated to be 4.12 × 10^−14^ cm^2^ s^−1^, higher than that of MnO/NC (1.05 × 10^−14^ cm^2^ s^−1^), suggesting faster reaction kinetics and enhanced Li-ion diffusion for CoO/MnO/NC.

**Figure 5:**
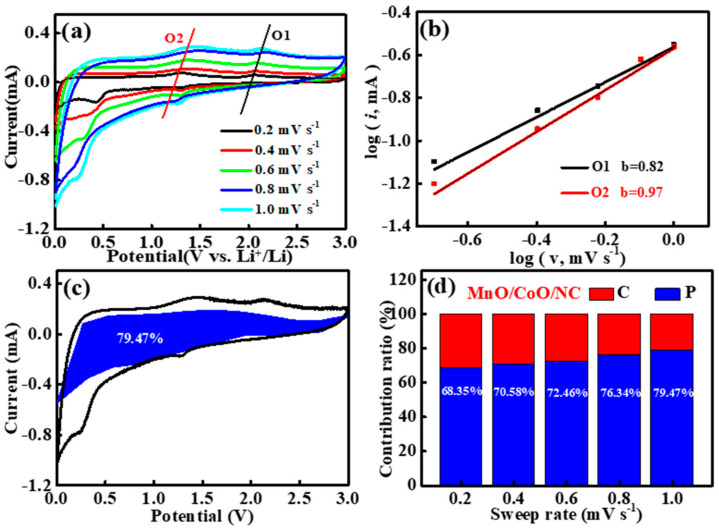
(**a**) CV curves of CoO/MnO/NC at different scan rates. (**b**) Log (i) vs. log (v) plots at each redox peak of CoO/MnO/NC. (**c**) Capacitive contribution to the total capacity of CoO/MnO/NC at 1 mV s^−1^. (**d**) The capacitance contribution percentage of CoO/MnO/NC at different scan rates.

The cyclic voltammetry (CV) curves obtained at different scan rates offer valuable insights into the capacity contribution and Li-ion diffusion kinetics of CoO/MnO/NC, as depicted in Figure 5a. Capacitive and diffusion-controlled contributions to the overall lithium storage behavior are discerned by examining the relationship of peak current and the scan rate (*i* = a*v*^b^), where a is the empirical parameter, and b is the contribution of the two parts to the stored charge, which can be obtained from the log (i) versus log (v) plots of CoO/MnO/NC at the oxidation peaks (O1 and O2). Electrochemical storage provides a primarily capacitive contribution when the b value is close to 1, whereas a value closer to 0.5 suggests dominant diffusion behavior [49,50]. The b values of CoO/MnO/NC electrode are 0.82 and 0.97 (Figure 5b), indicating that the capacitive contribution is dominant during the lithium storage process. The overall current (*i*) could be divided as capacitive (*k_1_v*) and diffusion-controlled current responses (*k*_2_*v*^1/2^), and it can be further quantitatively calculated using the following equation (3) [51,52]:

*i* = *k*_1_
*v* + *k*_2_
*v*^1/2^ (3)

where *v* is the scan rate. Quantitative analyses revealed that the capacitive contributions accounted for 79.47% of the overall charge storage capacity of CoO/MnO/NC at a scan rate of 1 mV s^−1^, as shown in Figure 5c. The capacitive contributions of CoO/MnO/NC were calculated to be 68.35%, 70.58%, 72.46%, 76.34%, and 79.47% (Figure 5d) at scan rates with a range of 0.2, 0.4, 0.6, 0.8, and 1 mV s^−1^, respectively. These values exceeded those of MnO/NC (Figure S7), underscoring the superior rate capability of CoO/MnO/NC, and the high capacitive contribution of CoO/MnO/NC heterostructures may be due to the abundant mesopores structures, improved electronic conductivity, and the fast electrochemical kinetics [53,54].

### 2.3. Theoretical Calculations

**Figure 6:**
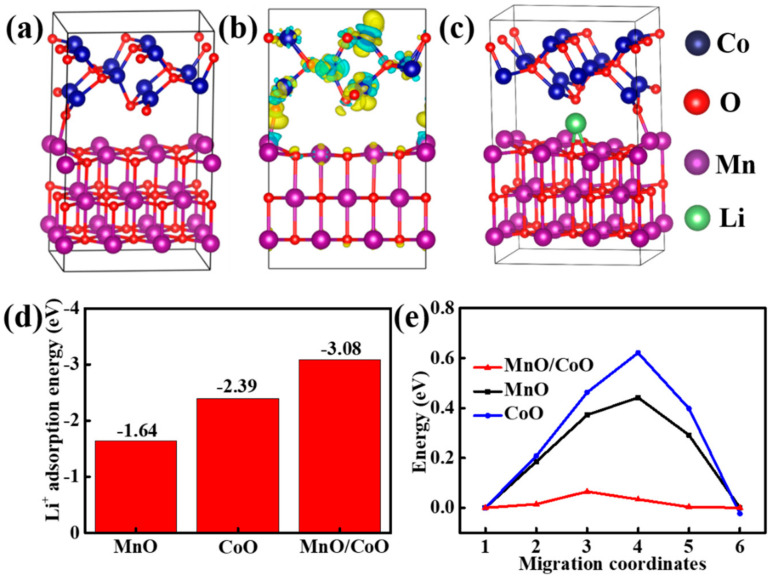
(**a**) Optimized structure, (**b**) charge density differences, and (**c**) optimized structure of CoO/MnO with a Li atom adsorbed in the interlayer. (**d**) The adsorption energy of CoO/MnO, MnO, and CoO. (**e**) Migration energy profiles for Li^+^ diffusion in CoO/MnO, MnO, and CoO.

The density functional theory (DFT) calculation was also performed to explore the effect of heterojunction on the electronic structure modulation of CoO/MnO. The crystalline structures of CoO and MnO are predicted in Figure S8, and the CoO (220) plane of CoO and (200) plane of MnO were selected to optimize the structure of the CoO/MnO model, as shown in Figure 6a. The charge density differences and optimized Li-ion adsorption configuration on CoO/MnO are shown in Figure 6b,c. Notably, a significant charge redistribution is observed at the heterogeneous interface, indicating spontaneous electron transfer from CoO to MnO and the consequent formation of an interfacial electric field and suggesting the presence of unique diffusion channels within CoO/MnO [23,55]. The total density of states (TDOSs) of CoO/MnO, MnO, and CoO results are presented in Figure S9, and the Fermi levels of all sample models are situated within the conduction bands, indicating their metallic nature and high electrical conductivity [56]. However, CoO/MnO shows the obvious and additional continuous states compared to MnO and CoO, suggesting that the construction of an interfacial electric field in CoO/MnO enhances electronic conductivity and facilitates efficient electron transfer [57,58,59]. Figure 6d shows the calculated Li adsorption energy of MnO, CoO, and CoO/MnO. CoO/MnO displays the lowest Li adsorption energy (−3.08 eV) compared to MnO (−1.64 eV) and CoO (−2.39 eV), indicating the strong Li-ion capture ability and favorable Li-ion adsorption in CoO/MnO [60]. Figure 6e exhibits the migration energy profiles of MnO, CoO, and CoO/MnO, and the value of CoO/MnO is lower than that of MnO and CoO, indicating faster Li^+^ diffusion in the CoO/MnO heterostructures [56,61]. Overall, these results indicate that the formed heterostructures of CoO/MnO can effectively improve electrochemical kinetics via electron structure regulation, in turn improving electrochemical performance.

## 3. Materials and Methods

### 3.1. Synthesis of CoO/MnO/NC

All raw materials were purchased from Shanghai Macklin Biochemical Technology Co., Ltd., Shanghai, China. Typically, 1.2 g of polyacrylonitrile (PAN) powders was firstly dissolved in 10 mL N,N-dimethylformamide (DMF) with vigorous stirring for 12 h at room temperature. Then, 0.49 g of Mn(Ac)_2_·4H_2_O, 0.125 g of Co(Ac)_2_·4H_2_O, and 0.6 g of Pluronic F127 were dissolved in a PAN-DMF solution with continuous stirring for 6 h, forming the precursor solution. During the electrospinning process, the precursor solution was transferred into a 5 mL plastic syringe equipped with a flat needle, and the working voltage was set as 18 kV. The injection speed (10 µL min^−1^) was controlled by a syringe pump, and aluminum foil was used as the collector. After the electrospinning process, the collected sample (Co-Mn PAN polymer nanofibers) was pre-oxidized and stabilized at 230 °C for 3 h in a drying oven under the air atmosphere. Finally, the as-functionalized carbon nanofibers were obtained by calcining the precursor film at 800 °C for 2 h under the nitrogen atmosphere, and it was noted as CoO/MnO/NC. The CoO/MnO/NC with different Mn/Co ratios was also prepared by controlling the amount of Mn(Ac)_2_·4H_2_O and Co(Ac)_2_·4H_2_O. For comparison, the pristine MnO/NC and CoO/NC nanofibers were also synthesized using the same method.

### 3.2. Theoretical Calculations

Density functional theory (DFT) calculations were employed in our work using the Vienna Ab inito Simulation Package (VASP) within generalized gradient approximation (GGA) with Perdew–Burke–Ernzerhof (PBE) functionals [62,63]. The nucleus–electron interaction was described using the projector augmented wave (PAW) potentials, and the dispersion interactions were described using Grimme’s DFT-D3 scheme [64,65]. A plane wave basis set energy cut-off of 500 eV was employed in the calculation. For the optimization of atoms and geometries, the relaxed convergence was set to be 1 × 10^−5^ eV for the total energy and 0.01 eV Å^−1^ for atomic forces.

### 3.3. Characterization

X-ray diffraction (XRD, Bruker D8, Bruker Company, Billerica, MA, USA) with Cu Kα radiation (λ = 1.5405 Å) was used to characterize the crystal structure of as-prepared electrode materials. The Raman spectrum was recorded using an Invia Raman spectrometer (LabRAM Aramis, HORIBA Jobin-Yvon Company, Paris, France). X-ray photoelectron spectroscopy (XPS) measurements were carried out using a photoelectron spectrometer (Thermo Scientific ESCALAB 250Xi, Thermo Fisher Scientific, Waltham, MA, USA) with an Al Kα radiation source. A scanning electron microscope (SEM, Sigma 500, Zeiss Company, Oberkochen, Germany) was used, and transmission electron microscopy (TEM, JEM-F200, JEOL, Akishima-shi, Japan) was performed to characterize the morphology and microstructure of the as-prepared products. The Brunauer–Emmett–Teller (BET) theory was employed when using a Belsorp max surface area detection instrument.

### 3.4. Electrochemical Measurements

The lithium storage performance of the as-prepared electrode materials was surveyed using CR2032 coin cells, and they were assembled in an argon-filled glove box. The working electrode was prepared using active materials comprising conductive carbon black and polyvinylidene fluoride with a mass ratio of 8:1:1, which were mixed with N-Methyl-2-pyrrolidone (NMP) and uniformly pasted on a Cu foil current collector, followed by drying in a vacuum at 120 °C for 12 h. Then, a 1 mol L^−1^ LiPF_6_ solution in a 1:1:1 mixture volume ratio comprising ethylene carbonate (EC), ethylene carbonate (DEC), and ethyl methyl carbonate (EMC) was used as the electrolyte, and lithium foil was used as the counter. The cells were charged and discharged on a Neware testing system at 0.01–3 V. CV, and EIS tests were conducted on a Parstat 4000+ electrochemical workstation (Princeton Applied Research, Princeton, NJ, USA).

## 4. Conclusions

In conclusion, a straightforward electrostatic spinning technique was used to synthesize CoO/MnO/NC electrode materials with heterostructures, and the CoO/MnO/NC electrode displays notable electrochemical properties, including a high reversible specific capacity of 936 mA h g^−1^ at 0.1 A g^−1^ after 200 cycles and an impressive high-rate capacity of 560 mA h g^−1^ at 5 A g^−1^. Both theoretical calculations using DFT and experimental data converge to highlight the exceptional performance of CoO/MnO/NC, elucidating intrinsic and extrinsic synergistic effects. (1) The large surface area and mesoporous structure can facilitate increased electrolyte contact, effectively mitigating volume expansion during cycling. (2) The heterostructures of CoO/MnO/NC can accelerate the electron/charge transfer and reduce the Li adsorption energy via electron structure regulation. (3) The enhanced electronic conductivity, coupled with the abundant mesoporous structure of CoO/MnO/NC, fosters a substantial capacitive contribution, thereby enhancing overall electrochemical efficiency.

## Data Availability

Data are contained within the article and Supplementary Materials.

## Supplementary Materials

The following supporting information can be downloaded at: https://www.mdpi.com/article/10.3390/molecules29102228/s1. Figure S1: SEM images of MnO/NC and CoO/NC; Figure S2: SAED pattern of CoO/MnO/NC with complex polycrystalline diffraction spots that corresponds to the MnO and CoO phase; Figure S3: (a) XPS survey spectrum and (b) high-resolution XPS spectras for O 1s of CoO/MnO/NC; Figure S4: Comparison of the cyclic performances of CoO/MnO/NC with different Mn/Co ratios at 0.1 A g^−1^; Figure S5: Comparison of the cyclic performances of CoO/MnO/NC with different Mn/Co ratios at 0.1 A g^−1^; Figure S6: Comparison of the cyclic stability of CoO/MnO/NC, MnO/NC and CoO/NC; Figure S7: (a) CV curves of MnO/NC at different scan rates. (b) Log (i) vs. log (v) plots at each redox peak of MnO/NC. (c) Capacitive contribution to the total capacity of MnO/NC at 1 mV s^−1^. (d) The capacitance contribution percentage of MnO/NC at different scan rates; Figure S8: The crystalline structure of CoO (a) and MnO (b); Figure S9: Calculated TDOS of CoO/MnO, MnO and CoO.

## Nomenclature

Item parameters	Units
Capacity	mA h g^−1^
Current density	A g^−1^
Voltage	V
Electron volt	eV
Scan rate	mV s^−1^
Diffusion coefficient	cm^2^ s^−1^
Resistance	Ω
Mass	g
Concentration	mol L^−1^
Temperature	°C
Time	h
Volume	mL
Per cent	%
Current	mA
Atomic forces	eV Å^−1^
