# Supplementary material for: Synergistic Engineering of CoO/MnO Heterostructures Integrated with Nitrogen-Doped Carbon Nanofibers for Lithium-Ion Batteries
Source: Molecules. 2024 May 9;29(10):2228. doi: 10.3390/molecules29102228 (PMC11123785; doi:10.3390/molecules29102228)
Supplement: Supplementary file 1 [file molecules-29-02228-s001.zip › molecules-2997046-supplementary.pdf]

Figure S1 SEM images of MnO/NC and CoO/NC.

Figure S2 SAED pattern of CoO/MnO/NC with complex polycrystalline diffraction spots that corresponds to the MnO and CoO phase.

Figure S3 (a) XPS survey spectrum and (b) high-resolution XPS spectras for O 1s of CoO/MnO/NC.

Figure S4 Comparison of the cyclic performances of CoO/MnO/NC with different Mn/Co ratios at 0.1 A g^{-1}.

Figure S5 The galvanostatic discharge/charge voltage profiles of CoO/MnO/NC at 0.1 A g^{-1}.

Figure S6 Comparison of the cyclic stability of CoO/MnO/NC, MnO/NC and CoO/NC.

Figure S7 (a) CV curves of MnO/NC at different scan rates. (b) Log (i) vs. log (v) plots at each redox peak of MnO/NC. (c) Capacitive contribution to the total capacity of MnO/NC at 1 mV s^{-1}. (d) The capacitance contribution percentage of MnO/NC at different scan rates.

Figure S8 The crystalline structure of CoO (a) and MnO (b).

Figure S9 Calculated TDOS of CoO/MnO, MnO and CoO.

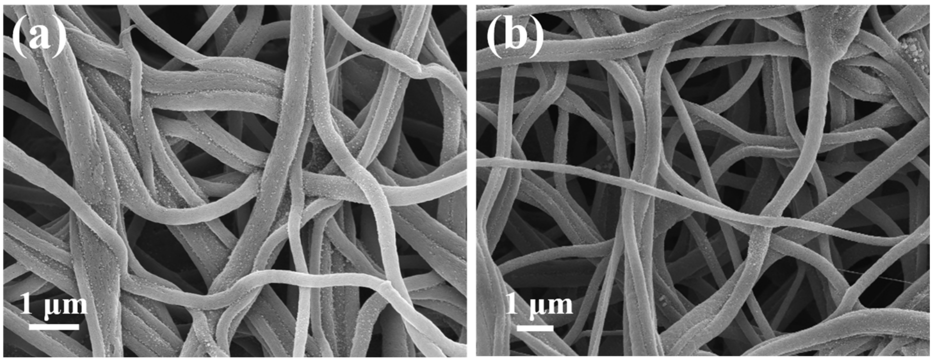

Figure S1 SEM images of MnO/NC and CoO/NC.

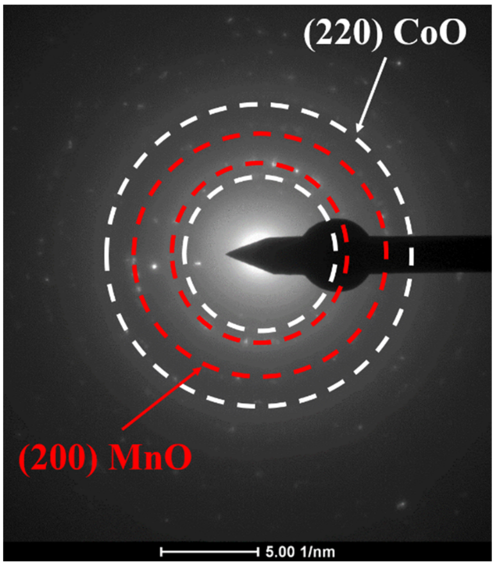

Figure S2 SAED pattern of CoO/MnO/NC with complex polycrystalline diffraction spots that corresponds to the MnO and CoO phase.

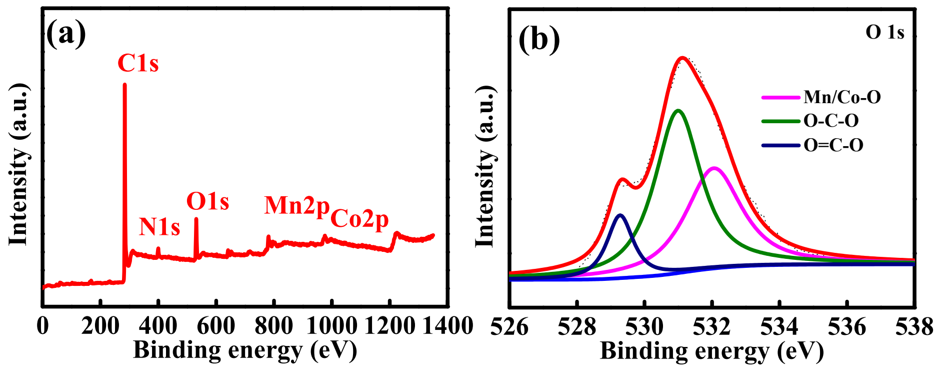

Figure S3 (a) XPS survey spectrum and (b) high-resolution XPS spectras for O 1s of CoO/MnO/NC.

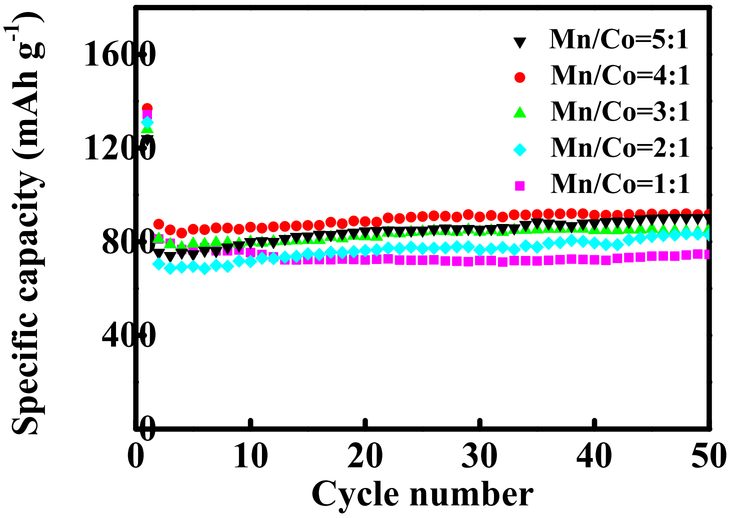

Figure S4 Comparison of the cyclic performances of CoO/MnO/NC with different Mn/Co ratios at 0.1 A g^{-1}.

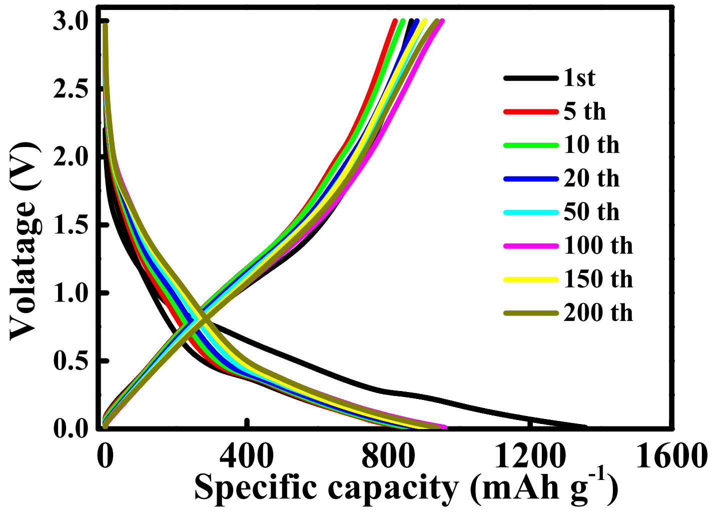

Figure S5 The galvanostatic discharge/charge voltage profiles of CoO/MnO/NC at 0.1 A g^{-1}.

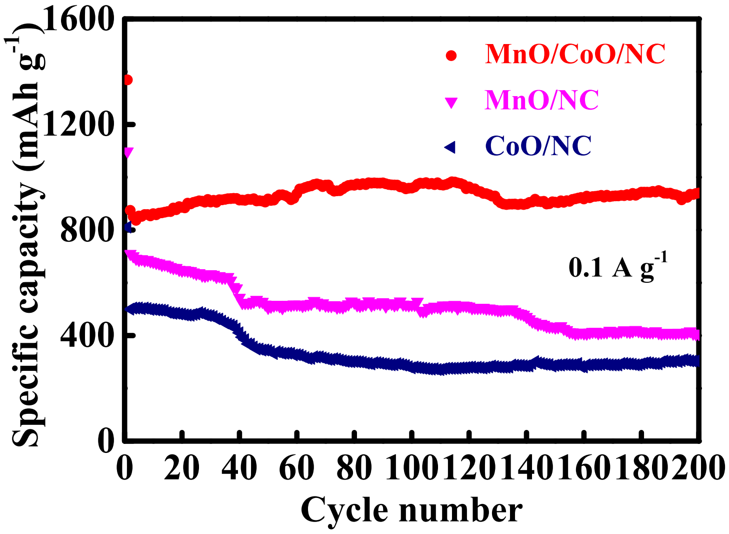

Figure S6 Comparison of the cyclic stability of CoO/MnO/NC, MnO/NC and CoO/NC.

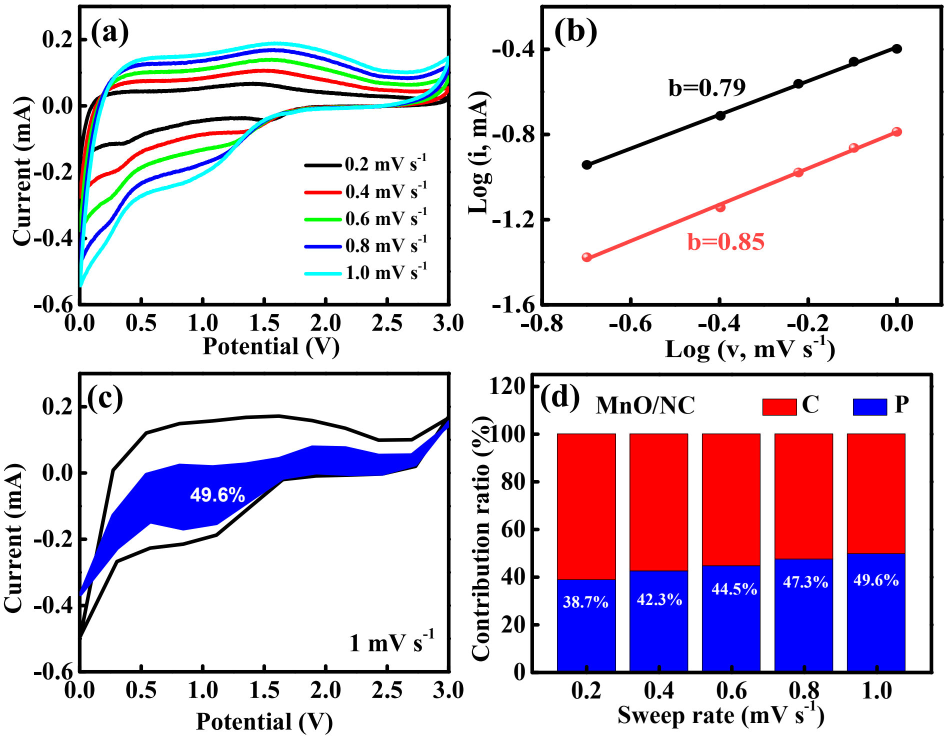

Figure S7 (a) CV curves of MnO/NC at different scan rates. (b) Log (i) vs. log (v) plots at each redox peak of MnO/NC. (c) Capacitive contribution to the total capacity of MnO/NC at 1 mV s^{-1}. (d) The capacitance contribution percentage of MnO/NC at different scan rates.

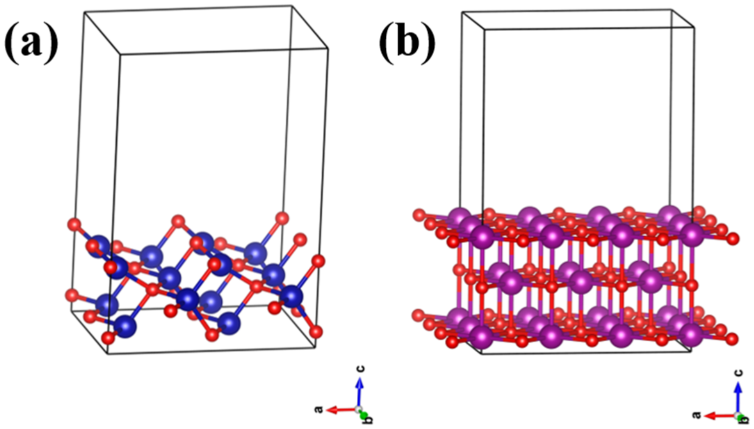

Figure S8 The crystalline structure of CoO (a) and MnO (b).

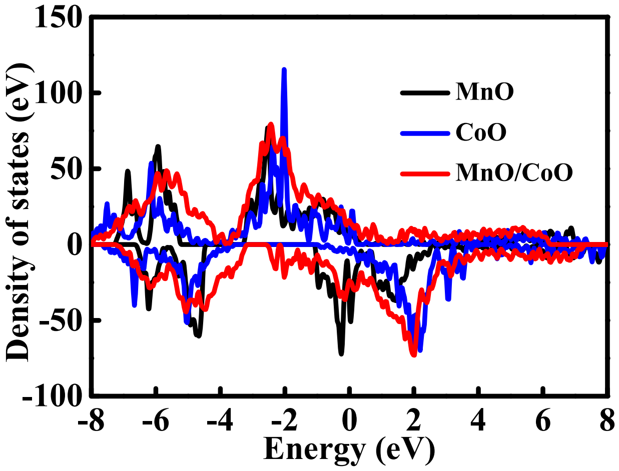

Figure S9 Calculated TDOS of CoO/MnO, MnO and CoO.
